# Data-driven and equation-free methods for neurological disorders: analysis and control of the striatum network

**DOI:** 10.3389/fnetp.2024.1399347

**Published:** 2024-08-07

**Authors:** Konstantinos Spiliotis, Rüdiger Köhling, Wolfram Just, Jens Starke

**Affiliations:** ^1^ Institute of Mathematics, University of Rostock, Rostock, Germany; ^2^ Laboratory of Mathematics and Informatics (ISCE), Department of Civil Engineering, Democritus University of Thrace, Xanthi, Greece; ^3^ Oscar-Langendorff-Institute of Physiology, Rostock University Medical Center, Rostock, Germany

**Keywords:** network physiology, equation free method, complex network dynamics, obsessive compulsive disorders, control of neurological disorders

## Abstract

The striatum as part of the basal ganglia is central to both motor, and cognitive functions. Here, we propose a large-scale biophysical network for this part of the brain, using modified Hodgkin-Huxley dynamics to model neurons, and a connectivity informed by a detailed human atlas. The model shows different spatio-temporal activity patterns corresponding to lower (presumably normal) and increased cortico-striatal activation (as found in, e.g., obsessive-compulsive disorder), depending on the intensity of the cortical inputs. By applying equation-free methods, we are able to perform a macroscopic network analysis directly from microscale simulations. We identify the mean synaptic activity as the macroscopic variable of the system, which shows similarity with local field potentials. The equation-free approach results in a numerical bifurcation and stability analysis of the macroscopic dynamics of the striatal network. The different macroscopic states can be assigned to normal/healthy and pathological conditions, as known from neurological disorders. Finally, guided by the equation-free bifurcation analysis, we propose a therapeutic close loop control scheme for the striatal network.

## 1 Introduction and context

Complex dynamical systems of interacting units appear in nature across several disciplines. Examples of these systems are networks of coupled neurons in the brain, epidemiological networks of interacting individuals during a virus spreading, and social or economic networks of human action and perception. A common characteristic of these networks is the existence of well-defined rules for each individual entity, the so-called microscopic description, while the emergent network behaviour evolves on a different level, the macroscopic scale.

The macroscopic description, say, in the form of ordinary or partial differential equations, governs the time evolution of few macroscopic variables, which are often given by low order statistics such as densities or correlation functions. It is however very challenging, if possible at all, to derive such a macroscopic description from a microscopic model, without making assumptions about the connectivity of the system, see, e.g., (Kevrekidis and Samaey, 2009; Montbrió et al., 2015). In neuroscience, and specifically for brain networks, the microscopic description is based on the electrochemical activity of individual cells which is frequently modelled by Hodgkin-Huxley equations (Hodgkin and Huxley, 1952; Terman et al., 2002; Spiliotis et al., 2022b). These cell-neurons interact through synaptic connections, and the mathematical description results in large systems of coupled nonlinear differential equations. The heterogeneous connectivity, the nonlinear behaviour of each cell, and the stochastic environment are factors which increase the complexity of the emergent network behaviour. Existence of multiple stationary states, sustained oscillations (Deco et al., 2008; Spiliotis and Siettos, 2011; Deco et al., 2013), as well as travelling waves and spatio-temporal chaos (Laing and Chow, 2002; Bhattacharya et al., 2022; Palkar et al., 2023), are signatures of the rich nonlinear behaviour of neural networks at the macroscopic level (Deco et al., 2008; Spiliotis and Siettos, 2011; Crowell et al., 2012; Deco et al., 2013; de Santos-Sierra et al., 2014; Siettos and Starke, 2016; Spiliotis et al., 2022b).

In previous studies (Spiliotis et al., 2022a; Spiliotis et al., 2022b; Spiliotis et al., 2024) we developed a large-scale computational model of the basal ganglia network and thalamus to describe movement disorders and treatment effects of deep brain stimulation. The model of this complex network covers three areas of the basal ganglia region: the subthalamic nucleus, the globus pallidus, both pars externa and pars interna, and the thalamus and motor and pre-motor cortex. Macroscopic analysis of the network dynamics allowed us to study the differences in neural activation patterns that will emerge within the brain’s structural network when simulating different medical conditions. For example, our computational model suggests that spatio-temporal activity in the basal ganglia network shows travelling wave solutions with more varying structures in the normal state as compared to the Parkinsonian state, see (Spiliotis et al., 2022b). In addition, the macroscopic analysis yields optimal frequency ranges for deep brain stimulation as well as optimal positions for the electrodes (Spiliotis et al., 2022a).

In this work, we focus on the striatum, an essential intermediate area of the brain that connects cortical to deep brain regions. The striatum belongs to the basal ganglia area and orchestrates activities for controlling movement, decision-making, choosing actions, and those maximising reward and other psychological behaviours (Calabresi et al., 2007; Crittenden and Graybiel, 2011; Calabresi et al., 2014). The striatum integrates cortical signals to create motor activities based on experience and forthcoming selections. The significance of striatum functionality is also accentuated by its involvement in a vast number of neurological diseases ranging from Parkinson’s disease, Huntington’s disease, and dystonia to psychological disorders such as obsessive-compulsive disorder, depression, impulsivity, and attention-deficit hyperactivity disorder (Remijnse et al., 2006; Crittenden and Graybiel, 2011).

Our main aim is the development of a mathematical-computational framework to analyse the macroscopic network behaviour of the striatum area, using data from microscopic simulations of a modified Hodgkin-Huxley network of neurons. We achieve our goal by an equation-free approach (Gear et al., 2005; Kevrekidis and Samaey, 2009; Marschler et al., 2014a; Laing, 2018). We identify the mean synaptic activity as the appropriate macroscopic variable that captures the network dynamics. This is also justified from other computational and medical-clinical studies Popovych and Tass (2019); Buzsáki (2004); Parasuram et al. (2016) since neural network activity like synchronisation, is also reflected by the amplitude of the local field potential (LFP) which is modelled as an ensemble-averaged synaptic activity of neurons. The equation-free method allows to perform a numerical bifurcation and stability analysis for the macroscopic dynamics. Our analysis will reveal an interesting property which is not accessible by straightforward simulations of the network, namely, the existence of two macroscopic network states, a high activation state and an unstable low activation state. The different macroscopic states can be related to healthy and pathological conditions existing in neurological disorders. During obsessive-compulsive disorder there is an increased cortico-striatal activity (Maltby et al., 2005; Marsh et al., 2014). Our computational model also predicts this high activation solution. Additionally the model shows a second solution which provides a low activation state, leading the striatum activity to a less pathological activation. Such a state is a potential healthy target for deep brain stimulation and may result in strategies for an efficient treatment. In fact, based on our analysis we propose a closed loop macroscopic control scheme which provides better performance compared to a straightforward deep brain stimulation approach.

## 2 Construction of the striatum model

**FIGURE 1:**
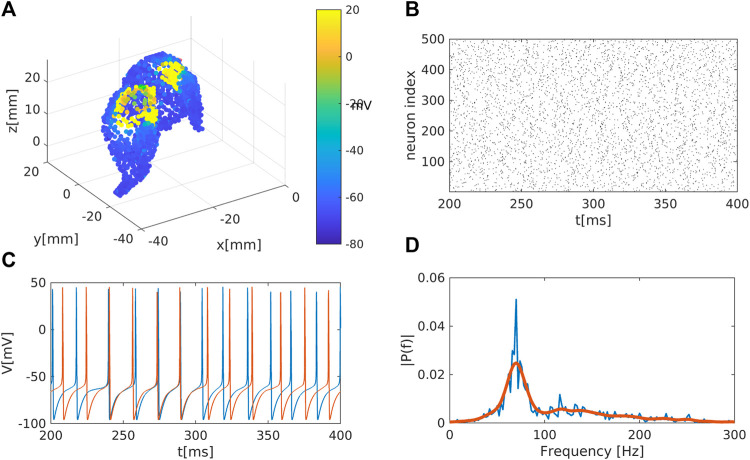
Representation of the striatal network: **(A)** Schematic representation of the striatum area as obtained in a MNI coordinate space. Colour code represents the membrane electrical activity in mV. **(B)** Raster plot representation of the network activity in time (in ms) and space (index of neuron of the nucleus). Black dots represent activated neurons (i.e., time dependent action potentials passing through −15 mV to positive values) **(C)** Time series of two representative medium spiny neurons (MSN) of the striatum. **(D)** Fourier analysis for the mean membrane activity $\bar{V}\left(t\right)=1/N\sum_{i}V_{i}\left(t\right)$ showing a γ rythm, i.e., with main characteristic frequency above 30Hz (such rhythm appears, for instance, in the striatum during motivated behaviour and reward processing (Kalenscher et al., 2010)).

We extract the surface of the striatum using magnetic resonance medical data taken from a previously published atlas (Iacono et al., 2015) and transform into the MNI (Montreal Neurological Institute) coordinate system. We place neurons randomly inside this area, see Figure 1A.

### 2.1 Modelling the striatum network by small word connectivity

We place in total 1995 neurons as network nodes in the striatum area. In line with medical studies (Yager et al., 2015) we assume that the vast majority of nodes (i.e., 95% of nodes) represent medium spiny neurons (MSN) while the remaining 5% of nodes are interneurons. The actual connectivity of the striatum is constructed following the idea of the small-world algorithm (Watts and Strogatz, 1998; Bassett and Bullmore, 2006; Stam and Reijneveld, 2007; Bullmore and Sporns, 2009; Spiliotis and Siettos, 2011). Small-world structures suitably model physiological networks (Netoff et al., 2004; Bassett and Bullmore, 2006; Bassett and Bullmore, 2017; de Santos-Sierra et al., 2014; Berman et al., 2016; She et al., 2016; Fang et al., 2017) since those networks are highly clustered and typically show short path lengths, enhancing in this way signal or rhythm propagation within the network and support synchronisation. Initially, each MSN is connected with k=20 neurons in the local neighbourhood of 5 mm diameter. Then, with a small probability p , for each local connection a new remote neighbour is added. For interneurons we follow the same approach, using, however, a five times higher interneuron to MSN connectivity with k=100 links. In this way, we obtain a network which is highly clustered with a small distance between nodes. The resulting striatal network gives a graph G=(V,E) , where V is the set of nodes and E the set of edges, i.e., the connections between neurons. The connectivity can be captured by a so-called adjacency matrix A , where $A_{ij}=1$ if there is a connection from node j to node i , and $A_{ij}=0$ otherwise. Each of the nodes represents a neuron with dynamics being described by modified Hodgkin-Huxley equations. The position of the striatum in the model is based on a medical atlas, and the positions of neurons are constructed based on this information. That means each index i comes with the Cartesian coordinates of the neuron and set of links. The connectivity of the neurons is constructed using the Watts and Strogatz small-world algorithm. In this type of connectivity, the nearest neurons are connected, and their activity is communicated to the nearest nodes, analogous to a graph Laplacian. Additionally, the small-world connectivity allows rare remote connections with a small probability, offering a more realistic neuronal network activity. As our model contains the actual geometric information of the position of neurons, we are finally able to model deep brain stimulation, where the position of electrodes and their spatial interaction with neighbouring neurons becomes essential (see, e.g., Eq. 13). It is the purpose of an equation-free method to reduce such a complex realistic description of the striatum to as few degrees of freedom as possible, by keeping the important dynamical signatures.

### 2.2 Modelling of the neuron dynamics

Our striatum network contains two types of neurons, the medium spiny neurons (MSN) representing 95% of all neurons and fast spiking neurons (FSI) which are the remaining ones. For the equations of motion of the neuron dynamics we follow (Chartove et al., 2020). It is reported therein, using models as well as experimental works, that striatal projection neurons (MSN) are capable of generating β oscillations. In contrast, striatal fast-spiking interneurons (FSIs) are responsible for generating delta and theta rhythmicity (at 2–6 Hz). In this sense, the FS-neurons are somewhat paradigmatic for GABAeric interneurons in the striatum (that means, neurons which use neurotransmitter gamma-aminobutyric acid in synapses, mainly to inhibit other neurons), although, obviously, other types such as Somatostatin-expressing inhibitory interneurons (SOM+) exist (Melzer et al., 2017). We chose to model the striatum mainly with parvalbumin-positive fast spiking interneurons (PV+) (Melzer et al., 2017). On the one hand they are among the best characterised neurons (Tepper et al., 2010). On the other hand a recent study focusing on identifying interneurons in the striatum found that those neurons accounted for the largest group of interneurons overlapping with 5HT3-EGFP, the marker which turned out to best identify interneurons but otherwise did not very much overlap with classical markers (Muñoz-Manchado et al., 2016). In addition PV+ are found more prominently in the dorsal, whereas SOM+ are more prominently found in the ventral striatum (Zandt et al., 2024). Finally Cholinergic neurons, in turn, act only via metabotropic receptors and hence slower than the GABAergic ones.

An MSN or FS neuron at node i is modelled by current balance equations for the membrane potential $V_{i}$

$$C\frac{dV_{i}}{dt}=-I_{\text{LEAK}}-I_{\text{K}}-I_{\text{Na}}-I_{\text{M/D}}-I_{\text{syn}}+I_{\text{app}}$$ (1)

where C is the membrane capacity. The current balance Eq. 1 contains four membrane currents, the fast sodium and potassium currents $I_{\text{Na}}$ , $I_{\text{K}}$ , the leak current $I_{\text{LEAK}}$ . For MSN neurons an M-current $I_{\text{M}}$ occurs whereas FS neurons contain a D-current $I_{\text{D}}$ (Chartove et al., 2020). All currents follow the Hodgkin Huxley formalism Hodgkin and Huxley (1952), that means $I_{X}=g_{X}m_{X}^{n_{1}}h_{X}^{n_{2}}\cdot \left(V-E_{X}\right)$ , where X∈{Na, K, M, LEAK} . The exponents $n_{1}$ , $n_{2}$ represent the number of activation-inactivation channels respectively, $g_{X}$ is the maximum conductance of the ion and $E_{X}$ stands for the reverse potential for each ion. Specifically, the sodium current has three activation gates and one inactivation gate so that

$$I_{\text{Na}}=g_{\text{Na}}m_{\text{Na}}^{3}h_{\text{Na}}\cdot \left(V_{i}-E_{\text{Na}}\right).$$ (2)

The potassium current has the form

$$I_{\text{K}}=g_{\text{K}}m_{\text{K}}^{4}\cdot \left(V_{i}-E_{\text{K}}\right).$$ (3)

and the leak current reads

$$I_{\text{LEAK}}=g_{\text{LEAK}}\cdot \left(V_{i}-E_{\text{LEAK}}\right).$$ (4)

Finally, the M- and D-current which enter the MSN and the FS neurons, respectively, are given by

$$I_{\text{M}}=g_{\text{M}}m_{\text{M}}\cdot \left(V_{i}-E_{\text{K}}\right),\;I_{\text{D}}=g_{\text{D}}m_{\text{D}}^{3}h_{\text{D}}\cdot \left(V_{i}-E_{\text{D}}\right).$$ (5)

The gating variables $m_{\text{Na}}$ , $h_{\text{Na}}$ , $m_{\text{K}}$ , $m_{\text{M}}$ , at node i each obey, following the Hodgkin-Huxley formalism (Hodgkin and Huxley, 1952; Chartove et al., 2020), an equation of the type

$$\frac{dx_{i}}{dt}=a_{x_{i}}\left(V_{i}\right)-\left(a_{x_{i}}\left(V_{i}\right)+b_{x_{i}}\left(V_{i}\right)\right)x_{i},$$ (6)

where $x_{i}\in \left\{m_{\text{Na}},h_{\text{Na}},m_{\text{K}},m_{\text{M}}\right\}$ stands for the respective gating variable at node i . The voltage dependent coefficients for the gating variables of the sodium current are given by

$$a_{m_{\text{Na}}}\left(V\right)=0.32\frac{V+54}{1-e^{-\left(V+54\right)/4)}},\;b_{m_{\text{Na}}}\left(V\right)=0.28\frac{\left(V+27\right)}{e^{\left(V+27\right)/5}-1}$$

and

$$a_{h_{\text{Na}}}\left(V\right)=0.128e^{-\left(V+50\right)/18},\;b_{h_{\text{Na}}}\left(V\right)=\frac{4}{1+e^{-\left(V+27\right)/5}}.$$

The coefficients of the activation gating for the potassium current read

$$a_{m_{\text{K}}}\left(V\right)=0.032\frac{V+52}{1-e^{-\left(V+52\right)/5)}},\;b_{m_{\text{K}}}\left(V\right)=0.5e^{-\left(V+57\right)/40},$$

and for those of the M-current we have

$$a_{m_{\text{M}}}\left(V\right)=0.032\frac{V+52}{1-e^{-\left(V+52\right)/5)}},\;b_{m_{\text{M}}}\left(V\right)=0.5e^{-\left(V+57\right)/40}.$$

The fast spiking neurons (FS) follow similar equations (Chartove et al., 2020), where instead of the M-current we use fast-activating, slowly inactivating D-current $I_{\text{D}}$ , given in Eq. 5, with three activation gates and one inactivation gate, thus imposing a delay in firing upon depolarisation (Golomb et al., 2007; Chartove et al., 2020). Table 1 contains the respective parameter settings for both types of neurons.

**TABLE 1:** Values for the conductance $g_{X}$ and inverse potential $E_{X}$ for the MSN and FS neurons.

Parameters $g_{X}$ and $E_{X}$	MSN	FS
$g_{\text{LEAK}}$	$0.1\text{mS}/{\text{cm}}^{2}$	$0.25\text{mS}/{\text{cm}}^{2}$
$g_{\text{K}}$	80 $\text{mS}/{\text{cm}}^{2}$	$225\text{mS}/{\text{cm}}^{2}$
$g_{\text{Na}}$	100 $\text{mS}/{\text{cm}}^{2}$	$112.5\text{mS}/{\text{cm}}^{2}$
$g_{\text{M}}$	1.3 $\text{mS}/{\text{cm}}^{2}$	-
$g_{\text{D}}$	-	$0.1\text{mS}/{\text{cm}}^{2}$
$E_{\text{LEAK}}$	−67 mV	−70 mV
$E_{\text{K}}$	−100 mV	−90 mV
$E_{\text{Na}}$	50 mV	50 mV
$E_{\text{M}}$	−100 mV	-
$E_{\text{D}}$	-	−90 mV

The current $I_{\text{app}}$ in Eq. 1 is written as $I_{\text{app}}=I_{0}+I_{\text{DBS}}$ , where $I_{0}$ predominantly represents a network activation current which describes the dependence of the neuronal activation due to intensity of cortical-striatal connectivity. The coupling between the neurons in Eq. 1 is described by the synaptic current $I_{\text{syn}}$ . Details will be outlined in the next Section 2.3. Since our network model contains realistic spatial details about the actual neural system we would be able to model the impact of deep brain stimulation as well. Thus, the term $I_{\text{DBS}}$ representing the deep brain stimulation, enters here as an additive contribution. In our analysis we keep $I_{\text{DBS}}=0$ , except the last section where we discuss the implementation of DBS in our model.

### 2.3 Description of the network inhibitory synaptic activity

We model the activation of a synapse using the activation variable $s_{i}$ for the i -th neuron (Compte et al., 2000; Laing and Chow, 2002; Ermentrout and Terman, 2012)

$$\frac{ds_{i}}{dt}=\alpha_{X}\left(1-s_{i}\right)H_{X}\left(V_{i}\right)-\beta_{X}s_{i},$$ (7)

where X∈{M,F} denotes whether the i -th neuron is a medium spiny neuron (M) or an interneuron (F), and $H_{X}\left(V\right)$ is a sigmoid function. The variable $s_{i}$ describes the activation of synapses from the pre-synaptic neuron i to post-synaptic neurons. The parameters $\alpha_{X}$ and $\beta_{X}$ in Eq. 7 determine the activation and inactivation time scales, respectively, of the inhibitory synaptic connections. For MSN we choose, following (Chartove et al., 2020),

$$H_{\text{M}}\left(V\right)=1+\tanh\left(V/4\right),$$

with activation rates $\alpha_{\text{M}}=2$ and $\beta_{\text{M}}=1/13$ . Similarly, for interneurons the expressions are given by

$$H_{\text{F}}\left(V\right)=1+\tanh\left(V/10\right),$$

with activation rates $\alpha_{\text{F}}=4$ and $\beta_{\text{F}}=1/13$ . For a neuron i of type X∈{M,F} the total synaptic inhibition it receives from pre-synaptic neurons of type Y∈{M,F} is given by

$$I_{i,\text{GABA}}=g_{\text{X}\text{Y}}\left(V_{i}-E_{\text{GABA}}\right)\underset{j\in Y}{\sum }A_{ij}s_{j},$$ (8)

where $A_{ij}$ is the adjacency matrix of the graph, the summation j∈Y is taken over neurons of type Y and $E_{\text{GABA}}=-80\text{mV}$ . The parameter $g_{\text{X}\text{Y}}$ represents the conductance between X and Y interactions with X,Y∈{M,F} .

The synaptic current for the MSNs consists of two parts, first the sum of synaptic currents over medium spiny neurons (describing the inhibition between MSN-MSN neurons) and second, the sum over interneurons (interneurons inhibition of MSN), so that Eq. 8 yields

$$I_{\mathrm{syn}}=g_{\text{MM}}\left(V_{i}-E_{\text{GABA}}\right)\underset{j\in \text{M}}{\sum }A_{ij}s_{j}+g_{\text{MF}}\left(V_{i}-E_{\text{GABA}}\right)\underset{j\in \text{F}}{\sum }A_{ij}s_{j}.$$ (9)

Similarly for an interneuron the synaptic current is given by

$$I_{\mathrm{sys}}=g_{\text{FF}}\left(V_{i}-E_{\text{GABA}}\right)\underset{j\in \text{F}}{\sum }A_{ij}s_{j}+g_{\text{FM}}\left(V_{i}-E_{\text{GABA}}\right)\underset{j\in \text{M}}{\sum }A_{ij}s_{j}.$$ (10)

Here the first sum represents the rare case of FS-FS inhibition, while the second term governs the feedback inhibitory loop of MSN to interneurons. For the conductivity values we use $g_{\text{MM}}=g_{\text{MF}}=0.02$ and $g_{\text{FF}}=g_{\text{FM}}=0.005$ .

In summary, Eqs 1, 2, 3, 4, 6, 7, 9, and 10 constitute a high dimensional heterogeneous set of coupled nonlinear differential equations defined on a graph with adjacency matrix A . The state of each neuron at node i is described by the set of variables $\left(V_{i},{\left(m_{\text{Na}}\right)}_{i},{\left(h_{\text{Na}}\right)}_{i},{\left(m_{\text{K}}\right)}_{i},{\left(m_{\text{M}}\right)}_{i},s_{i}\right)$ . Figures 1B,C illustrates the temporal dynamics of the network.

## 3 Equation-free method for analysing macroscopic network behaviour

To describe the main idea in basic terms, consider a high-dimensional dynamical system, for instance the dynamics of the neural network presented in the previous section. The network model evolves in time under specified known microscopic rules, e.g., the equations of motion for each node described above. Denote by $U_{t}\in R^{N}$ the state of the full network. Its time evolution over a time interval T is given by

$$U_{t+T}=\Phi_{T}\left(U_{t}\right)$$

where the so-called flow $\Phi_{T}:R^{N}\to R^{N}$ can be obtained from the (numerical) integration of the microscopic equations of motion.

We are interested in analysing the network behaviour on a different macroscopic scale. Assume there exists a suitable low-dimensional macroscopic variable $x\in R^{d}$ with d≪N , which captures the emergent collective behaviour of the network dynamics. Such collective coordinates $x_{t}=R\left(U_{t}\right)$ depend on the degree of freedoms of the system, and are determined by a restriction map $R:R^{N}\to R^{d}$ . Often one uses suitable averages for the purpose to capture the dynamics of a system at a macroscopic scale, see, e.g., (Kozma et al., 2005; Kevrekidis and Samaey, 2009; Spiliotis and Siettos, 2011). The motion of the macroscopic variable per time step is then given by

$$x_{t+T}=R\left(\Phi_{T}\left(U_{t}\right)\right)=\left(R◦\Phi_{T}\right)\left(U_{t}\right).$$

The main assumption of the Equation-Free approach (Gear et al., 2003; Kevrekidis and Samaey, 2009; Marschler et al., 2014a; Sieber et al., 2018) is that a macroscopic description in the form of closed equations of motion for the collective coordinate $x_{t}$ exists, even if one is unable to provide an analytic derivation for such effective low-dimensional equations of motion. The success of this idea relies on the ability to construct a meaningful lifting operator $L:R^{d}\to R^{N}$ such that

$$U_{t}=L\left(x_{t}\right)$$

gives for each macrostate $x_{t}$ a particular microstate $U_{t}$ which represents all the possible microstates which are consistent with $x_{t}$ . As with respect to the restriction map, the lifting operator needs to fulfil the obvious consistency constraint R◦L=I where I denotes the identity map. Whether such a lift exists depends of course on the microscopic model, the emergence of collective motion, and a suitable choice for the macroscopic variable. If all these conditions are met one obtains the macroscopic evolution law

$$x_{t+T}=R\left(\Phi_{T}\left(L\left(x_{t}\right)\right)\right)=\left(R◦\Phi_{T}◦L\right)\left(x_{t}\right)=F_{T}\left(x_{t}\right),$$

and the equations of motion, i.e., $F_{T}$ , can be derived from a numerical integration of the microscopic equations of motion over short time intervals T , without the need of going through cumbersome calculations (hence the notion of an equation-free approach). If successful, one can finally utilise algorithms to compute dynamical signatures, such as fixed points and their stability, as well as complete bifurcation diagrams from the macroscopic description in terms of $F_{T}$ , see for instance (Gear et al., 2003; Kevrekidis and Samaey, 2009; Siettos, 2011). An implicit equation-free analysis (Marschler et al., 2014b; Sieber et al., 2018) could further minimise the numerical errors in the computed bifurcation diagram.

## 4 Equation-free analysis of the striatum model

In this section, we apply the theoretical framework of an equation-free approach (Gear et al., 2005; Kevrekidis and Samaey, 2009; Marschler et al., 2014a) to analyse the emergent network dynamics macroscopically. Initially, we describe the lifting and restriction operator as well as the timestepper construction. Then, we discuss the consequences of the resulting one-dimensional evolution equation.

### 4.1 Lifting and restriction operator

The mean synaptic activity of MSNs turns out to be a suitable macroscopic variable

$$S_{t}=\frac{1}{N}\underset{i\in \text{M}}{\sum }s_{i}\left(t\right).$$ (11)

While such a choice looks appealing, and can be justified with hindsight, one can also support this choice by a more subtle data analysis using for instance diffusion maps, a data-driven method for dimensional reduction and manifold learning (Coifman and Lafon, 2006; Nadler et al., 2006; Laing et al., 2010; Marschler et al., 2014a; Dsilva et al., 2018). Here we skip those technical details and take Eq. 11 as our reduction map.

The crucial step to build the timestepper is the lifting operator. The construction of the lifting operator is based on two steps. First, we record a microscopic realisation of the system from a previous simulation, i.e., we store all the microscopic variables after a short period of 20 ms. Then, in the second step, we assign synaptic variables to the 1856 MSNs in the following way: Given a mean synaptic activity $S_{t}$ , we assign synaptic variables to the 1856 MSNs by randomisation, $s_{i}\left(t\right)=S_{t}+0.05\cdot Z_{i}$ where $Z_{i}∼N\left(0,1\right)$ are uncorrelated normal random variables, while we keep the other microscopic degrees of freedom unchanged. Then, we numerically integrate the coupled Hodgkin-Huxley equations for all neurons for a (macroscopically) short time T , to derive the new network microstate $U_{t+T}=\Phi_{T}\left(L\left(S_{t}\right),I_{0}\right)$ . The time T is chosen such that the other variables become enslaved to the mean synaptic activity $S_{t}$ . In fact, relatively short bursts on short time scales establish such a slaving relation (Gear et al., 2005; Spiliotis and Siettos, 2011). Constructing heuristically a lifting operator which results in a microscopic initialisation closer to the unknown attractive slow manifold leads to a faster convergence to the macroscopic behaviour and smaller numerical errors of the explicit equation-free analysis. As already mentioned, an implicit equation-free analysis (Marschler et al., 2014b; Sieber et al., 2018) could even further minimise the numerical errors in the computed bifurcation diagram.

**FIGURE 2:**
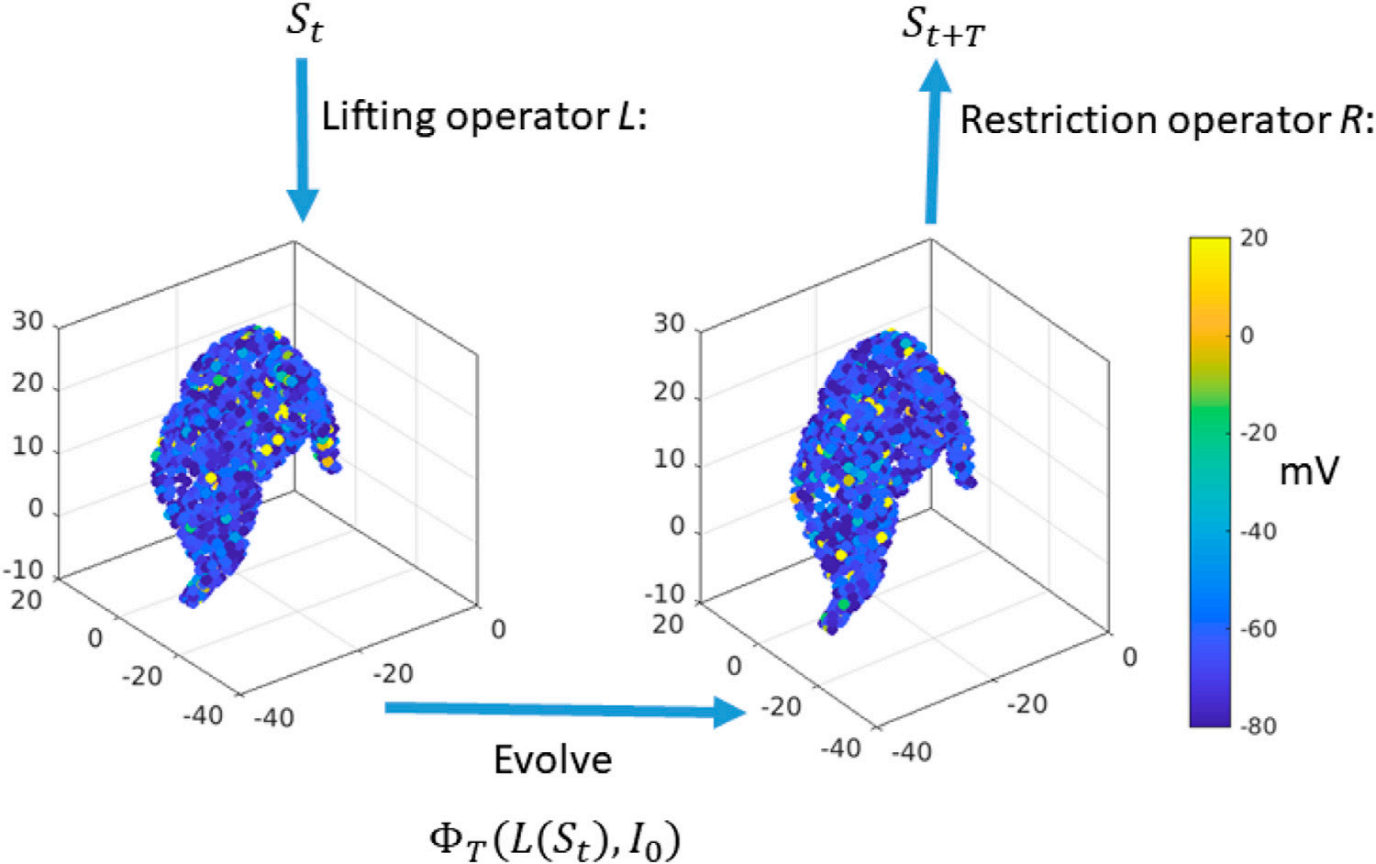
Equation-free construction of the timestepper: Start with the macroscopic variable $S_{t}$ , the mean synaptic activity. Transform this value into a consistent microscopic network state $U_{t}\in R^{n}$ through a lifting operator. We simulate the network equations for the all neurons and for a short macroscopic time T to derive the new network microstate $U_{t+T}$ . Finally, average the synaptic variables $s_{i}$ to obtain the macroscopic variable $S_{t+T}$ .

Finally, we apply the restriction operator to the new network microstate $U_{t+T}$ , i.e., we compute the mean synaptic activity $S_{t+T}$ which then defines the macroscopic evolution law $S_{t+T}=F_{T}\left(S_{t},I_{0}\right)$ . Here we have explicitly noted a constant network activation current $I_{\mathrm{app}}=I_{0}$ as a static parameter of our model (see Eq. 1). Figure 2 shows a graphical representation of the numerical procedure.

Since the macroscopic variable $S_{t}$ changes little on the time scale T we can approximate the time discrete dynamics by a time continuous first order differential equation

$$S^{\prime }\left(t\right)=f\left(S\left(t\right),I_{0}\right)\approx \frac{F_{T}\left(S_{t},I_{0}\right)-S_{t}}{T}.$$ (12)

where the right hand side $F_{T}\left(S_{t},I_{0}\right)$ in the difference quotient is determined by our equation free approach.

### 4.2 Data-driven system identification, stability and bifurcation analysis

**FIGURE 3:**
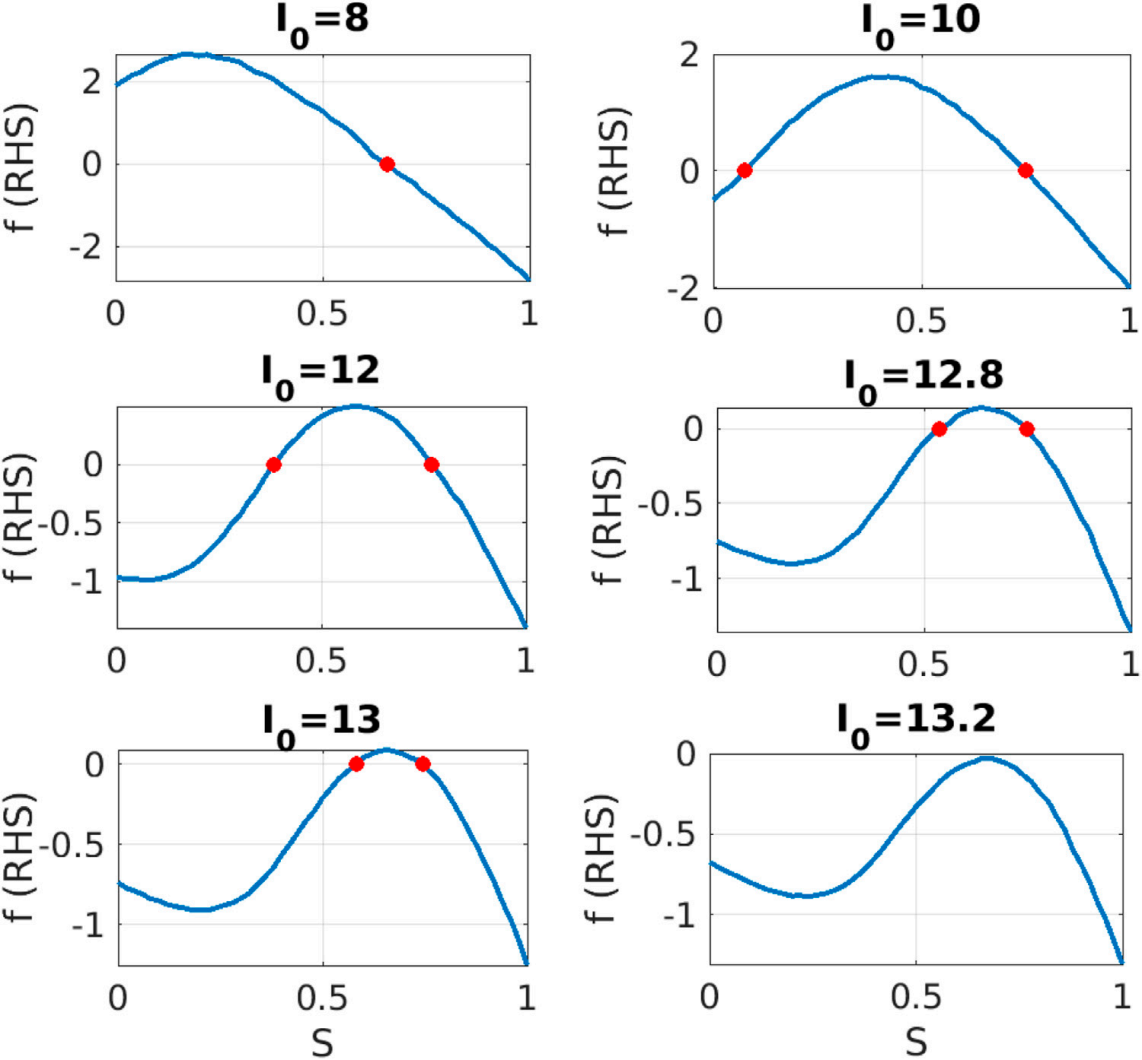
Equation-free system identification: For different values of parameter $I_{0}$ , we construct numerically the right hand side f in dependence on the mean synaptic activity S . Red dots indicate the zeros of f , i.e., the fixed point solutions of the macroscopic dynamics. Clearly, the right hand side shows one fixed point at $I_{0}=8$ , two fixed points in the range $I_{0}\in \left[10,13.2\right]$ and finally, no fixed point for $I_{0}>13.2$ .

Using Eq. 12 we can construct the right hand side f of the macroscopic equation of motion. We perform independent parallel computations by covering the phase space with an equidistant mesh of initial values for the macroscopic variable, and the $I_{0}$ axis with an equidistant lattice of parameter values. We thus obtain the right hand side $f\left(S,I_{0}\right)$ with fairly high numerical resolution. The results for f in dependence on S are depicted in Figure 3, for $I_{0}=8$ , 10, 12, 12.8, 13, and $I_{0}=13.2$ . Despite a quite noisy neuron dynamics we obtain a rather smooth result for f which shows little fluctuations. The computation of f has been based on macroscopic averages over at about 2000 neurons and an ensemble average of 20 realisations, resulting in statistical errors of about 0.5% , in line with the data shown in Figure 3. The fixed points of the macroscopic dynamics are given by the zeros of the function f , while the sign of the slope at the zero determines the stability of the fixed point. The fixed point is stable for negative slope, while the fixed point is unstable for positive slope. Here stability refers to stability with respect to the macroscopic dynamics which is solely governed by the mean synaptic activity S(t) . While the internal microscopic dynamics is still highly complex, at the macroscopic level the motion is captured by the single scalar quantity S(t) . With our equation free approach we have been able to determine the right hand side of the macroscopic equation of motion (22), see Figure 3. Thus, the zeros of this right hand side and the sign of the slope allow us to determine the location and the macroscopic linear stability of the macroscopic stationary state.

**FIGURE 4:**
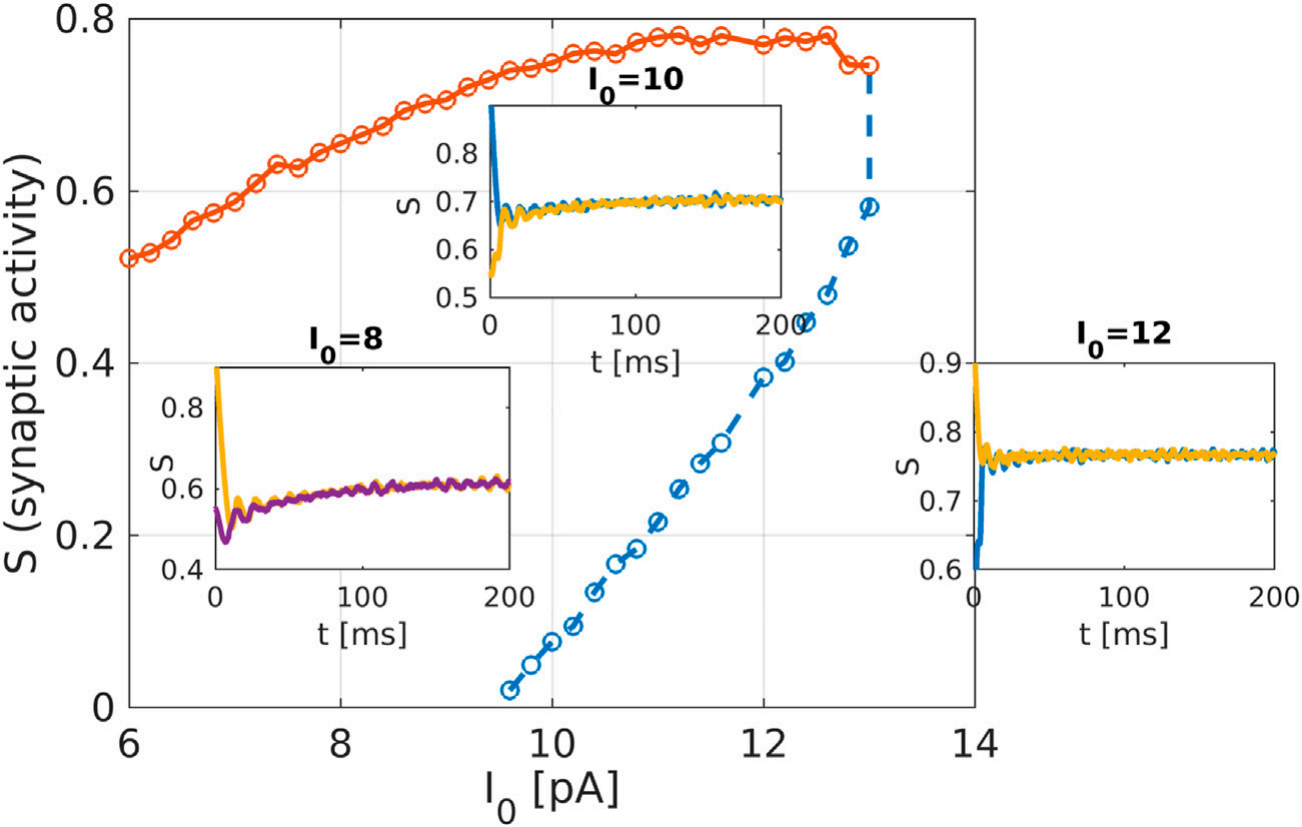
Macroscopic system analysis of the striatum network: Bifurcation diagram as obtained from the equation-free analysis of the striatum network. The network activation current $I_{\mathrm{app}}=I_{0}$ is used as the bifurcation parameter, and the mean synaptic activity of MSNs acts as macroscopic variable. Solid line (red) are stable fixed points, dashed line (blue) are unstable fixed points. The two branches disappear in a saddle-node bifurcation at $I_{\text{CRIT}}=13.19$ . The insets show temporal simulations of mean synaptic activity S, for $I_{0}=8,10,12$ . Simulations converge to the upper stable branch of the bifurcation diagram.

We observe that the shape of right hand side f is smooth and the graph shifts down, as the value of parameter $I_{0}$ increases. As a consequence the number of macroscopic fixed points changes. For $I_{0}=8$ we obtain one stable fixed point. As the value of $I_{0}$ increases, e.g., for $I_{0}=10$ , we see two fixed points, an unstable one at small values $S^{*}=0.09$ and a second stable one at $S^{*}=0.73$ with high network activation. For increasing values of the parameter $I_{0}$ these two fixed points still persist with stability properties unchanged, but at a critical value close to $I_{0}=13.2$ the two fixed points collide and disappear in a saddle-node bifurcation. We can condense this information in a bifurcation diagram, see Figure 4. There are two branches of steady state solutions. The high neural activation solutions are stable (solid red line in Figure 4) while the low activation branch is unstable (dashed blue line in Figure 4). These two branches bifurcate in a saddle node bifurcation at $I_{\text{CRIT}}=13.19$ . In general, an increased intensity of the current $I_{0}$ changes the rhythmicity and the density of activation. In the pathological case which corresponds to high activation, neurons exhibit spiking activity with variable periods (i.e., non-constant period between two spikes), and some neurons appear to show brief intervals of synchronised activity, preceded and followed by non-synchronous firing. Such synchrony could either be due to transient common activation via network inputs (e.g., inhibition of fast-spiking neurons), or it could actually occur by chance with this tonic firing at a relatively high frequency. The equation-free method remarkably reveals also an unstable low neural activation branch. Such an unstable state is not accessible by direct numerical simulations of the network model, it is a genuine outcome of the equation free approach. In terms of the microscopic dynamics such a state corresponds to an invariant saddle in the full phase space containing all microscopic degrees of freedom. For a potential neurophysiological interpretation of this state we recall that during the pathological case of obsessive-compulsive disorder, there is a hyperactivity of the striatum network. Thus, the stable high-activation branch of the solution can be seen as a pathological condition. The unstable low activation state that cannot be reached in direct simulations is nevertheless accessible by control techniques, such as closed loop deep brain stimulations. When successful, stabilising this unstable low activation state will produce a therapeutic effect on the striatum network hyperactivity.

## 5 Discussion

The recently invented field of network physiology aims at inferring dynamical interactions in complex biological or medical systems from observed data. With its inherently interdisciplinary intention this field aims to understand, based on data analysis, modelling approaches, or clinical practice, how diverse biological or physiological sub-systems interact from the cellular microscopic to the phenomenological macroscopic level, to explain diverse physiological phenomena, such as healthy or unhealthy states (see, e.g., (Schöll, 2022) for a recent editorial). Looking at the emerging field of network physiology from an equation free perspective has the potential to add an additional facet to this area of research. An equation free approach aims at uncovering the complex dynamical behaviour at a macroscopic level without the need to reconstruct the complex underlying microscopic dynamical network, thus addressing a main goal of network physiology from the outset. We have showcased a computational framework to analyse biophysical neuronal network models, and we applied the method to the striatum area. Based on a realistic mathematical model for the microscopic dynamics of the striatum we have been able to detect relevant macrostates and their dynamical features using an equation-free approach. One major contribution of this research work is that the method bridges the different levels of spatio-temporal scales, the microscopic ones where the physics of neurons is known and the macroscopic ones where the analysis is performed. The activity of neurons and the individual synaptic activity is given using the Hodgkin-Huxley formalism, which constitutes the microscopic description of the model. The network connects these neurons and produces a macroscopic or emergent behaviour with different spatio-temporal properties. Importantly, our equation-free approach allows us to study this emergent behaviour in detail, i.e., to perform stability and bifurcation analysis. The synaptic activity shows steady behaviour, which corresponds to the high network activity, the upper branch of solution in Figure 4, while the corresponding spectrum of the mean membrane activity shows a characteristic peak at the gamma band (see as well Figure 1D). Several other studies also analyse the macroscopic network activity or the emergent network behaviour (Fesce, 2024; Kromer and Tass, 2024; Venkadesh et al., 2024). Additionally, in (Kromer and Tass, 2024), a detailed study of mean synaptic activity, including synaptic plasticity, is performed. The proposed equation-free approach can be applied to these works containing multiple spatio-temporal scales. For example, by studying synaptic plasticity, one can extract critical values of synaptic strength, which contribute to phase transition in the macroscopic network dynamics (Marschler et al., 2014a).

Our realistic microscopic model was based on an FDA-approved state-of-the-art human atlas (Iacono et al., 2015) extracting coordinates for the striatal neurons, on modified Hodgkin-Huxley equations for medium spiny neurons (MSN) and fast-spiking neurons (FSN) (Hodgkin and Huxley, 1952; Chartove et al., 2020), and on complex network structures for neuronal connectivity (Netoff et al., 2004; Bassett and Bullmore, 2006; Bassett and Bullmore, 2017; de Santos-Sierra et al., 2014; Berman et al., 2016; She et al., 2016; Fang et al., 2017). Depending on the parameters, the network model produces patterns which can be associated with healthy or pathological conditions, reflected by low or high synaptic activity. In clinical studies of obsessive-compulsive disorder (Maltby et al., 2005; Marsh et al., 2014) a dysfunctional hyperactivity of the frontal-striatal circuits is observed similarly to the high activation state we obtain in our model for increasing the intensity of the cortico-striatal current $I_{0}$ .

Within an equation-free approach we were able to investigate the crucial macroscopic behaviour for the mean synaptic activity. Such an analysis not just reproduces the dynamically stable high activity branch, but also shows an unstable low activity state which is inaccessible by direct simulations of the model. Such unstable dynamical states could be promising targets for treating pathological conditions.

Deep brain stimulation (DBS) of the striatum has evolved as a promising therapy for patients with severe and resistant forms of obsessive compulsive disorders (OCD) and mental impairments (Rodriguez-Romaguera et al., 2012; Blomstedt et al., 2013; Widge et al., 2019; Wu et al., 2021). While there exist different computational approaches modelling DBS for OCD, see for instance (Szalisznyó and Silverstein, 2021), we can utilise our realistic large scale dynamical system to obtain insights about pathological neural activity during OCD. Since our model has been based on the realistic spatial structures of the striatum each neuron, labelled by an index i , comes with its corresponding spatial position, given by a vector ${\vec{x}}_{i}$ . The impact of the current applied in DBS and acting on the i -th neuron depends on the distance between the position of the electrode in real space, denoted by ${\vec{x}}_{E}$ , and the position of the i -th neuron. It is modelled by

$$I_{\text{DBS}}=A_{\text{DBS}}\exp\left(-\|{\vec{x}}_{i}-{\vec{x}}_{E}{\|}^{2}/\sigma^{2}\right)H\left(\sin\left(\omega_{\text{DBS}}t\right)\right)\cdot \left(1-H\left(\sin\left(\omega_{\text{DBS}}t+\delta_{\text{DBS}}\right)\right)\right.$$ (13)

**FIGURE 5:**
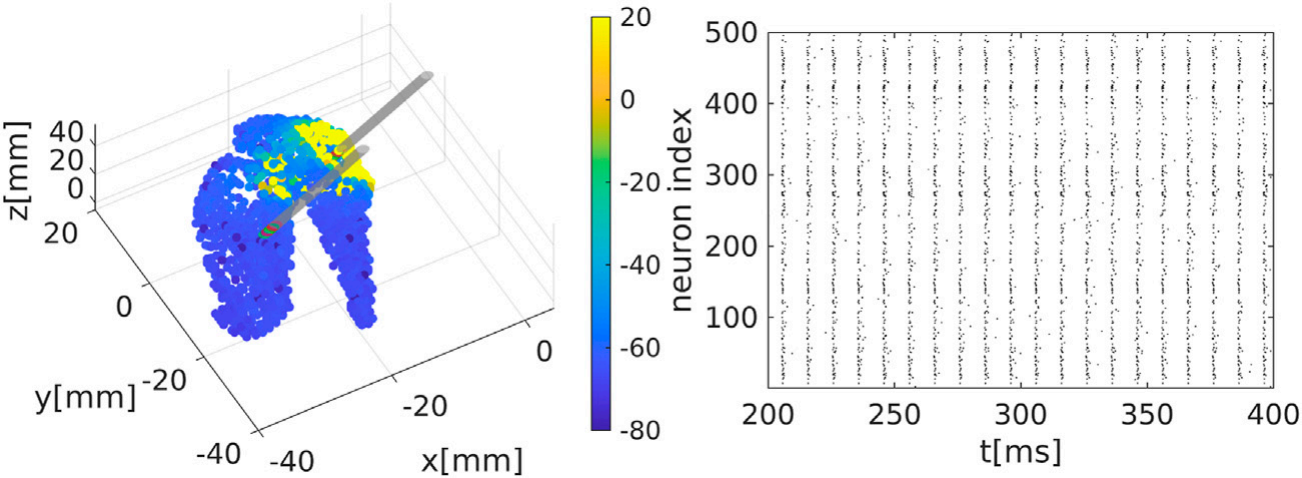
Deep brain stimulation (DBS) on the striatum model: Simulation of the network model with the current Eq. 13 added to the network equations.

This quantity enters the equation for the i -th neuron in an additive way, $I_{\text{app}}=I_{0}+I_{\mathrm{DBS}}$ , see Eq. 1. The impact of DBS decreases super-exponentially with respect to the distance from the electrode. Far from the electrode, the intensity current is almost zero, having no impact on the neuron action, and different electrode positions result in different network activations. Here $\omega_{\text{DBS}}=2\pi /T_{\text{DBS}}$ denotes the angular frequency, $T_{\text{DBS}}$ the period, and the phase shift $\delta_{\text{DBS}}$ the duration of the pulse, with H abbreviating the Heaviside function ( H(x)=1 , if x>0 , else H(x)=0 ). The function H takes into account that DBS is not applied as a plain harmonic, but in terms of periodic pulses. The crucial amplitude and frequency in DBS is determined by the two parameters $A_{\text{DBS}}$ and $\omega_{\text{DBS}}$ , respectively. Figure 5 shows the application of DBS at ${\vec{x}}_{E}=\left(-9,9,5\right)$ , with the DBS amplitude and frequency $A_{\text{DBS}}=200$ and $\omega_{\text{DBS}}=2\pi /130$ . We observe that DBS induces strong synchronisation in the neural activity of striatum.

**FIGURE 6:**
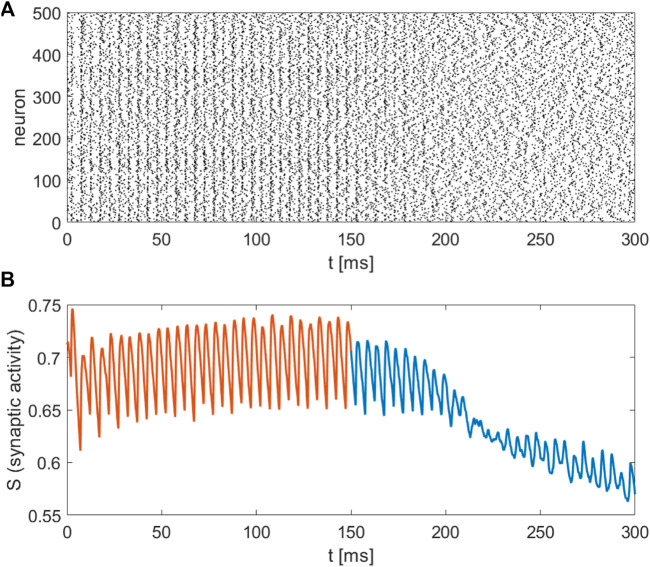
Closed loop control scheme for DBS on the striatum network: Application of DBS with constant amplitude along the line of Eq. 13 for t<150ms (red). Close loop control scheme for DBS, using Eq. 14, adjusting the DBS amplitude by linear proportional feedback for t>150ms . **(A)** Raster plot for n=500 randomly chosen neurons. Black dots represent activated neurons (i.e., time dependent action potentials passing through −15 mV towards positive values. **(B)** The mean synaptic activity for DBS without control (red), and DBS with linear proportional feedback (blue).

Thanks to the equation-free framework we are now able to design a macroscopic proportional feedback controller for DBS. For instance, for $I_{0}=10$ , the equation-free analysis showed the existence of one unstable fixed point at mean synaptic activity $S^{*}=0.08$ . We use the amplitude of DBS, that means $A_{\text{DBS}}$ , as control variable which is adjusted due to linear proportional feedback

$$\frac{dA_{\text{DBS}}}{dt}=-K_{p}\left(S-S^{*}\right)$$ (14)

where $K_{p}$ denotes the gain of the control. By choosing the gain appropriately we aim at driving the system towards the low activation state. Figure 6 shows the application of DBS at the point ${\vec{x}}_{E}=\left(-9,9,5\right)$ and for frequency 200Hz. We observe that after switching on the feedback control ( t>150 ms) the macroscopic activity gets closer to the healthy low activation state, see Figure 6B, and that synchronisation is destroyed in favour of a desynchronised state, see Figure 6A. In general, explaining the mechanism of DBS and how it acts in the evolved brain network is still a mystery. For example, in Parkinson’s disease, it is unclear whether DBS suppresses or enhances the neural activity of the targeted areas (Rubin and Terman, 2004; Montgomery and Gale, 2008). In Figure 6, we present two stages of DBS: the first 150 ms without a control scheme and the second part after 150 ms. While DBS without control induces synchronised activity of neurons such a synchronised state is suppressed when control is turned on. In that respect the closed-loop DBS results in realistic patterns closer to healthy conditions.

There are still considerable unknowns for a successful application of DBS such as the anatomical targets of stimulation, optimal stimulation parameters like amplitude and frequency of stimulation, as well as long-term effects of stimulation. In obsessive compulsive disorders hyperactive frontal-striatal activity has been reported (Maltby et al., 2005; Marsh et al., 2014). We conjecture that this hyperactivity is qualitatively similar to the stable upper branch solution as depicted in the bifurcation diagram Figure 4. Since our network model allows for properly modelling the network activation current a corresponding equation-free analysis of the model may then provide some answers to the open questions raised above. Our successful simple showcase provides evidence that such an ambitious program may succeed.

## Data Availability

The original contributions presented in the study are included in the article/supplementary material, further inquiries can be directed to the corresponding authors.
